# Socio-demographic Determinants of Household Food Insecurity among Iranian: A Population-based Study from Northwest of Iran

**Published:** 2018-06

**Authors:** Jafar Sadegh TABRIZI, Leila NIKNIAZ, Homayoun SADEGHI-BAZARGANI, Mostafa FARAHBAKHSH, Zeinab NIKNIAZ

**Affiliations:** 1. Tabriz Health Services Management Research Center, Faculty of Management and Medical Informatics, Tabriz University of Medical Sciences, Tabriz, Iran; 2. Tabriz Health Services Management Research Center, Tabriz University of Medical Sciences, Tabriz, Iran; 3. Road and Traffic Injury Research Center, Dept. of Statistics and Epidemiology, Faculty of Health, Tabriz University of Medical Sciences, Tabriz, Iran; 4. Research Center of Psychiatry and Behavioral Sciences, Tabriz University of Medical Sciences, Tabriz, Iran; 5. Liver and Gastrointestinal Diseases Research Center, Tabriz University of Medical Sciences, Tabriz, Iran

**Keywords:** Food insecurity, Family size, Educational level, Employment, Iran

## Abstract

**Background:**

We aimed to evaluate the household food security status and associated factors in East-Azerbaijan, Iran (urban and regional areas).

**Methods:**

Data (n=1385) as a part of the major lifestyle promotion project conducted in northwest of Iran were collected in 2015, by short form of the Household Food Security Scale consisting six questions. The Chi-square and Logistic regression were used to for statistical analysis.

**Results:**

The prevalence of food insecurity was 58.4%. The rate of food insecurity in the residents of capital city (59.7%) was higher than those of residents of regional cities (57.18%), however, this difference was not statistically significant (*P*=0.36). The respondents with family size more than 3 (*P*=0.01), unemployed (0.001) and married (0.01) respondents and the ones with lower education levels (*P*<0.001) were significantly more food insecure than other respondents. After adjusting for depending factors, the marital status, household size, educational level and the employment status of the head of the family had significant association with food security.

**Conclusion:**

Family size, employment status and educational level of the head of the family were significant predictors of food insecurity. Policymakers should focus on increasing minimum education levels and employment situations to decrease food insecurity.

## Introduction

Food insecurity is a serious public health problem because individuals’ health, well-being, and quality of life are linked to their household food security. Adults in food-insecure households have higher rates of chronic illnesses such as obesity, depression, diabetes, and heart disease (1–3). Furthermore, in the context of food insecurity the management of chronic diseases is also compromised (4, 5).

Food insecurity is a frequent problem in both developed and developing countries (6–8). Worldwide, owing to various social, economic and environmental disparities, more than 850 million people face food insecurity (9). While in the USA, 85.7% of American households were food secure throughout the entire year in 2013 (10), in South Africa only 48% of South African were food secure (11). Moreover, the correlates of food insecurity were investigated in different courtiers. In rural areas of Malaysia, household food insecurity was significantly correlated with household size, educational level and employment status (12). In Uganda, household size, educational level, and employment status were the main determinants of food insecurity (13).

In Iran, there have been major improvements over the last three decades in reducing food insecurity and Iran has had the greatest reduction in global hunger index (GHI) ranking in the Middle East. However, there were limited up-to-date studies in Iran, which tackle the issue of food security. In Tehran, about 16% of had low and very low food security status (14). In Ray City, Iran, the household food insecurity prevalence was 55% (15), in Yazd, 33% (16), Asadabadi region of Tabriz (North-west of Tabriz), 36.3% (8), and Arak, 73.4%, which had significant association with age, sex, educational level of the head of the family and the socioeconomic status of the family (17).

Most of the studies regarding prevalence of food insecurity were conducted in central cities of Iran and also limited data is available about the determinant of food insecurity in Iran. According to the importance of the effect of food security on health and quality of life, this study was conducted with the aim of determining the prevalence of food insecurity in East-Azerbaijan (north-west of Iran) and also investigating its association with various social-demographic characteristics.

## Materials and Methods

### Research design

Data were collected in 2015 as a part of the major lifestyle promotion project (LPP) conducted in the districts of East Azerbaijan (urban and regional parts), one of the largest provinces of Iran. This study was conducted by probability proportional to size (PPS) multistage stratified cluster sampling through which 150 clusters selected. In PPS sampling, the selection probability for each element was set to be proportional to its size measure. The sampling frame was based on the postal code frame of the national post office, updated yearly. The clusters were selected in this system based on postal code. Each address in this system was summarized in a 10-digit postal code number. In urban areas, clusters comprise one to several blocks or parts of blocks. Blocks were usually attached buildings.

For sample size calculation, based on predicted prevalence rates of 26% (8), confidence interval of 95%, study power of 80%, attrition rate of 20%, sample size was calculated to be 1320. However, 1500 people involved because of the variety of the measured variables.

After determining the cluster start point, enrollment and data collection was started. In each cluster, 10 participants were enrolled (1500 participants). This began from the household at the cluster start point and continued toward the other households until the required number of participants enrolled. Consecutive households were selected based on the geographical location of buildings to the right-hand side of each building. Research survey and examination teams visited households, according to previously arranged appointments.

Exclusion of incomplete questionnaire yielded 1385 final sample, subjected to statistical analysis. The final sample consisted of 656 the capital city (Tabriz) residents and 717 regional (cities and rural areas) [including Marand, Mianeh, Varzegan, Khodafarin, Bonab, Osku and Ilkhichi] residents. All procedures performed in this study were in accordance with the ethical standards of the Ethics Committee of Tabriz University of Medical Science (registration number: 1394.383) and with the 1964 Helsinki declaration and its later amendments or comparable ethical standards. Moreover, informed consent was obtained from all individual participants included in the study.

### Instrumentation

The research data were collected through two questionnaires through face-to-face interviews. The first questionnaire included questions regarding the socio-demographic characteristics, including age, gender, educational level, marriage status and residential area. The second questionnaire was the short form of the Household Food Security Scale consisting six questions (Table 1). This questionnaire was validated internationally (18, 19) in the local language previously (8). Responses of “often” or “sometimes” on questions Q5 and Q6, “yes” on Q1, Q3 and Q4 are coded as affirmative (yes). Responses of “almost every month” and “some months but not every month” on Q2 are coded as affirmative (yes). The sum of affirmative responses to the six questions in the module is the household’s raw score on the scale if the participants affirmed the two or more of the six items, they were considered food insecure.

**Table 1:** Demographic characteristics of the participants (n=1385)

	***N***	**%**
**Age (yr)**		
15–24	194	14.00
25–34	382	27.58
35–44	353	25.48
45–54	256	18.48
55–65	200	14.44
**Family size**		
<3	617	44.54
>3	768	54.45
**Gender (%)**		
Male	659	47.58
Female	726	52.41
**Marital status**		
Single	171	12.34
Married	1214	87.65
**Education**		
Illiterate	197	14.227
Undergraduate	963	69.53
college	225	16.24
**Residency area**		
Urban	675	48.73
regional	710	51.26
**employment status**		
employed	700	50.54
unemployed	50	3.61
housewife	635	45.84

### Data Collection

Data was collected based on interviews by a team of trained health professionals. Research survey and examination teams visited households according to previously arranged appointments. All participants were interviewed to obtain socio-demographic characteristics and the food security questions.

### Statistical analysis

SPSS (ver. 18, Chicago, IL, USA) software was used for all statistical analyses. The demographic characteristics represented the independent variables and presented as number and percent (%). Chi-square was conducted to compare the food security status considering gender, age, marital status and residential area. The logistic regression test was used to investigate the association between food security status and demographic factors. A significance level of 0.05 was used.

## Results

Mean age of the participants was 39.47±13.05 years. Approximately, 52% of the participants were female, 16.24% of them had a college degree, 87.65% of them were married and 50.54% of them were employed. About 48.73 % of participant lived in the capital city (Tabriz) (Table 1).

Generally, 58.4% (809) of participants were food insecure and 41.6% (576) of them were food secure. About 61% of respondent could not afford to eat balanced diet (consists of all major food groups). Moreover, 50% of them affirmed that the food did not last and often did not have money to get more foods. About 40% of participants cut the size of meal or skip meals because of the lack of money for the food in the last 12 months. About 43% of the participants affirmed that they eat less than their felt because of the lack of money (Table 2).

**Table 2:** Affirmative responses to individual items on the short food security questionnaire ^a^

***Question***	***Number***	***(%)***
1. Did you ever cut the size of meals or skip meals because of lack of money for food in the last 12 months? (Yes, No)	579	40.4
2. If yes, how often? (Almost every month, some months but not every month, only 1 or 2 months)	540^b^	69.2
3. Did you ever eat less than you felt you should because there was not enough money to buy food in the last 12 months? (Yes, No)	595	43.0
4. Were you ever hungry but didn’t eat because you couldn’t afford enough food in the last 12 months? (Yes, No)	197	14.20
5. Food didn’t last and didn’t have money to get more. (Was that often, sometimes, or never true for you in the last 12 months?)	701	50.6
6. Couldn’t afford to eat balanced meals. (Was that often, sometimes, or never true for you in the last 12 months?)	847	61.1

a. “Yes” is the affirmative response to questions 1, 3, and 4; “almost every month” and “some months but not every month” are affirmative responses to question 2; “often” and “sometimes” are affirmative responses to questions 5 and 6. Two or more affirmative responses to the questions indicate food insecurity

b. total samples size for this item is 579 (responders who had affirmation response to item 1)

The food security status of respondents according to their socio-demographic status shows in Table 1. The 45–54 yr old responders, females, respondents with the family size more than 3, residents of the capital city, unemployed respondents and the ones with lower education levels were more food insecure than other respondents. The Chi-square test showed the significant differences in insecurity status of females (versus males), married (versus singles), respondents with household size >3 (versus household size<3), unemployed respondents (versus employed ones) and low educated respondents (versus higher educated ones).

Table 3 presents the logistic regression coefficient for the association between food security status and demographic factors. It is apparent from this table that after adjusting for depending factors, the household size, educational level and the employment status of the head of the family had significant association with food security. Respondents with households size of <3 were significantly more food secure than respondents with households size of >3 (regression coefficient 0.67 (0.51, 0.88)). Moreover, the respondents with unemployed the head of the family were significantly more food insecure than employed ones (regression coefficient 0.40 (0.19, 0.81)) (Table 4). Considering the educational level, the respondents with higher educational level (undergraduate and college level) were more food secure than lower educational level. Moreover, the single respondents were significantly more food secure than married ones.

**Table 3:** Food security status of participant according to their socio-demographic status

	***Food security status***		
	**Food secure NO (%)**	**Food Insecure NO (%)**	*P*-value^*^
**Total**	576 (41.6)	810 (58.4)	-
**Age (yr)**			
15–24	84 (43.29)	110 (56.70)	0.20
25–34	163(42.68)	219 (57.32)	
35–44	152 (43.05)	201 (56.93)	
45–54	99 (38.67)	157 (61.32)	
55–65	78 (39)	122(61)	
**Family size**			
<3	280 (45.38)	337 (54.61)	**0.01**
>3	296 (38.54)	472 (61.45)	
**Gender (%)**			
Male	291 (42.23)	368 (57.76)	**0.06**
Female	285 (39.25)	441 (60.74)	
**Marital status**			
Single	86 (50.29)	85 (49.70)	**0.01**
Married	490 (40.36)	724 (59.63)	
**Residency area**			
Urban	272 (40.29)	403 (59.70)	0.36
regional	304 (42.81)	406 (57.18)	
**employment status**			
employed or self-employed		379 (54.14)	**0.001**
unemployed	321 (45.80)	37 (74)	
housewife	13 (26)	393 (61.88)	**<0.001**
**Education**	242 (38.11)		
Illiterate	50 (25.38)	147 (74.61)	
Undergraduate	383 (39.77)	580 (60.22)	
college	143 (63.55)	82 (36.44)	

* *P*-value of chi-square //

*P*-value<0.05 is considered as a significance level

**Table 4:** Logistic Regression coefficient for the association between food security and demographic factors

	***B (95% CI)***	P-value^**^
**Age(yr)**		
15–24	1(Ref) ^*^	-
25–34	1.20 (0.79, 1.82)	0.37
35–44	1.54 (0.98, 2.40)	0.05
45–54	1.39 (0.86, 2.26)	0.17
55–65	1.45 (0.86, 2.44)	0.15
**sex**		
Male	1(Ref) ^*^	-
Female	0.94 (0.631, 1.392)	0.76
**Marital status**		
single	1 (Ref)^*^	-
Married	0.576 (0.36, 0.89)	**0.01**
**Family size**		
<3	1 (Ref) ^*^	-
>3	0.74 (0.58, 0.94)	**0.01**
**Number of Under 6 yr old child**		
0		
<3	1(Ref) ^*^	-
>3	0.90 (0.68, 1.21)	0.51
**Region**	0.60 (0.05, 7.02)	0.69
Regional	1 (Ref) ^*^	-
Urban	1.16 (0.938, 1.45)	0.18
**Employment status(head of family)**		
Employed	1(Ref) ^*^	-
Unemployed	0.38 (0.19, 0.76)	**<0.001**
Housewife	0.97 (0.65, 1.45)	0.907
**Educational level**		
Illiterate	1(Ref) ^*^	-
undergraduate	2.04 (1.395, 2.98)	**0.01**
college	5.17 (3.23, 8.27)	**<0.001**

Regression model was adjusted for age, Sex, marital, education, employment, and residency status, number of under 6-year-old children at home //

* ref: reference group //

** *P*-value<.05

## Discussion

According to the importance of food security in health and quality of life, the present study was designed to determine the prevalence and associated factors of food security status in East-Azerbaijan, Iran. Generally, of 1385 responders, about 58.3% (810) were food insecure. The prior study in one distinct of Tabriz, Iran reported the prevalence of food insecurity about 36.3% (8). The observed discrepancy between these results may be due to the timing of studies and differences in study population. The food security status of only one distinct of Tabriz was studied (Asadabadi Medical Centre) however; we included a large sample of the capital city and regional cities population of East-Azerbaijan province.

57.18% of surveyed regional households and 59.70% of capital city households were food insecure. A study on 7158 households (2496 rural and 4662 urban) in Iran observed 87% of rural households and 71% of urban households were food secure (20). In another study, 40.4% of studied rural households were food secure and 59.6% of them were food insecure in northwest of Iran (21). In northeast of Iran, 40.9% of surveyed rural households were food secure (22). Food insecurity in Tabriz City was higher than regional areas which may be due to high rate of immigration from rural areas to urban areas. This group may experience increasing rates of unemployment and poverty, disease, inadequate educational resources, and declining availability and/or accessibility to adequate health and social services

In the present study, food insecurity was associated with the number of household members, which is consistent with the results of other studies (13, 23, 24). The observed relationship between the household size and food insecurity is may be due to the fact that in situations, such as rise in the price of food or temporary joblessness, the bigger the household is the lower the amount of food each household member consumes.

In the term of the association between educational level and food security status, this study produced results which corroborate the findings of other studies in this field (24). The observed relationship between education level and food insecurity may be attributed to the fact that people with higher education have higher income and therefore can provide enough food much more easily than others.

Confirming those of another study in Tabriz (24), the result of the present study showed that food insecurity is negatively associated with the employment status of the head of the family. The employed head of the family can provide enough food for their family members much more easily than others. However; being a housewife was not a significant factor associated with household food insecurity. Contrary to this result, being a housewife was a significant factor associated with household food insecurity in Malaysia (12).

The main strength of the present study was a large sample size from different urban and regional areas that serve quite different populations. Besides, a validated questionnaire was used for measuring food security status. One of the main limitations of the present study that might influence the findings is the cross-sectional design of the study, which implies that no causal inferences could be made about the demographic determinants of food insecurity.

## Conclusion

As health authorities worldwide struggle to improve eating behaviors and food security, it is essential to better understand the drivers of these. Approximately 57.18% of regional cities respondents and 59.70% of urban responders were food insecure. Moreover, the family size, marital status, the employment status and the educational level of the head of the family were significant predictors of food insecurity. Therefore, policymakers should mainly focus on increasing minimum education levels while an increase in minimum wages may help low-income individuals avoid food insecurity. Additional prospective researchers were needed to determine the relationship between food security status and health-related conditions.

## Ethical considerations

Ethical issues (Including plagiarism, informed consent, misconduct, data fabrication and/or falsification, double publication and/or submission, redundancy, etc.) have been completely observed by the authors.
